# Dataset from a randomized controlled trial comparing intensive short-term dynamic psychotherapy to waitlist for treatment-resistant depression

**DOI:** 10.1016/j.dib.2025.112126

**Published:** 2025-10-07

**Authors:** Rasoul Heshmati, Frederik J. Wienicke, Ellen Driessen

**Affiliations:** a University of Tabriz, Faculty of Education and Psychology, Department of Psychology, 29 Bahman Boulevard, 5166616471 Tabriz, Iran; b Radboud University, Behavioural Science Institute, Department of Clinical Psychology, Thomas van Aquinostraat 4, 6500 HE Nijmegen, the Netherlands; c Pro Persona Mental Health Care, Depression Expertise Center, Nijmeegsebaan 61, 6525 DX Nijmegen, the Netherlands

**Keywords:** Intensive short-term dynamic psychotherapy, Negative affect, Emotional repression, Treatment-resistant depression, Randomized controlled trial

## Abstract

The data presented in this article concern 86 Iranian individuals with treatment-resistant depression (TRD), who participated in a randomized controlled trial comparing intensive short-term dynamic psychotherapy (ISTDP) to a waitlist condition. Adults with major depressive disorder (MDD), who had not responded to prior treatment with antidepressant medication, were randomly assigned to ISTDP (*n* = 43) or waitlist (*n* = 43). The dataset includes demographic variables and the outcome measures: all subscales of the Weinberger Adjustment Inventory (WAI), and the Negative Affect subscale from the Positive and Negative Affect Schedule (PANAS-NA). Outcome measures were collected at baseline, post-treatment (10 weeks), and 3-month follow-up.

Specifications Table

**Table:** 

Subject	Social Sciences
Specific subject area	Treatment-Resistant Depression, Psychotherapy.
Type of data	Table, SPSS file
Data collection	Data were collected from adults with TRD, recruited between April and May 2020 in Tabriz, Iran. Participants received either ISTDP or were assigned to a waitlist control condition between June and August 2020. Follow-up data were collected in November 2020. Eligibility for trial participation required meeting the Diagnostic and Statistical Manual of Mental Disorders (4th edition) criteria for MDD and prior antidepressant non-response. Individuals with severe psychiatric, cognitive, or medical conditions or recent psychotherapy were excluded. Farsi versions of the WAI and PANAS-NA were used to assess depression, emotional repression, and negative affect at baseline, post-treatment, and 3-month follow-up.
Data source location	University of Tabriz, Tabriz, Iran.
Data accessibility	Repository name: Open Science Framework (OSF) Data identification number: https://doi.org/10.17605/OSF.IO/75PU8 Direct URL to data: https://osf.io/wxg8p
Related research article	R. Heshmati, F.J. Wienicke, E. Driessen, The effects of intensive short-term dynamic psychotherapy on depressive symptoms, negative affect, and emotional repression in single treatment-resistant depression: A randomized controlled trial, Psychother. 60 (2023) 497–511. https://doi.org/10.1037/pst0000500.

## 1 Value of the Data

• This dataset can be valuable for researchers studying the efficacy of ISTDP, as well as depression treatment outcomes, emotional repression, and negative affect.
• The data can be reused in secondary analyses such as individual participant data (IPD) meta-analyses focused on depression treatment, and especially short-term psychodynamic interventions, for TRD.
• It offers a rare contribution from a non-Western, Middle Eastern clinical sample, supporting culturally diverse psychotherapy research.
• The inclusion of validated Farsi versions of psychological measures enables cross-cultural comparisons and supports psychometric research in underrepresented populations.
• Repeated measurement across three time points allows for investigation of symptom trajectories and short-term maintenance of treatment effects.

## 2 Background

Intensive Short-Term Dynamic Psychotherapy (ISTDP) is a brief, emotion-focused treatment that aims to reduce psychiatric symptoms by helping patients experience and process unconscious attachment-trauma-related emotions [[1], [2], [3]]. It differs from other (psychodynamic) therapies through its confrontational approach and emphasis on mobilizing complex transference feelings [4].

While previous studies have shown promising results for ISTDP in treating numerous disorders [[5], [6], [7]], evidence for its efficacy regarding treatment-resistant depression (TRD) [8,9] primarily stems from a single research group. Moreover, the effects of ISTDP on emotional repression and negative affect, two constructs central to ISTDP's theoretical model, remain underexplored [3].

This study aimed to independently replicate earlier findings regarding ISTDP for TRD and expand outcomes to include measures of emotional repression and negative affect. Moreover, the study offered insight into the application of ISTDP in a non-Western clinical context.

This data article complements the original publication [10] by describing the dataset and providing access to the full anonymized dataset via the Open Science Framework [11], thereby facilitating replication, contributing to IPD meta-analyses, and supporting further analyses of psychodynamic constructs in TRD.

## 3 Data Description

The dataset [11] contains information on 86 individuals and is provided in long format as an SPSS (.sav) file. It includes 37 variables, covering trial variables, helper variables, demographic and clinical characteristics (see Table 1 for summary statistics), and outcome measures. These variables are organized as follows:

• 4 trial variables: participant number, treatment condition, dropout, and therapist number;
• 10 helper variables: dummy codes for time and interaction terms;
• 6 demographic variables: age, gender, socioeconomic status, education level, marital status, and employment status;
• 2 clinical characteristics: number of previous antidepressant trials and current use of antidepressant medication;
• 15 outcome variables at three time points (baseline, post-treatment, and 3-month follow-up):
  ○ 14 variables from the Weinberger Adjustment Inventory (WAI):
    ▪ 3 scale scores (Distress, Restraint, and Defensiveness),
    ▪ 10 associated subscale scores (Distress: Anxiety, Depression, Low Self-Esteem, Low Well-Being; Restraint: Suppression of Aggression, Impulse Control, Consideration of Others, Responsibility; Defensiveness: Repressive Defensiveness, Denial of Distress)
    ▪ the Repressive/Restraint Composite Score
  ○ 1 variable from the Negative Affect and Positive Affect Schedule (PANAS): negative affect subscale score.

**Table 1:** Descriptive statistics of the sample.

Variable	*N* = 86
	M[SD]/n ( %)
Age	36.9 [11.7]
Gender	
Female	53 (61.6)
Male	33 (38.4)
Marital status	
Divorced/Widowed	10 (11.6)
Married	48 (55.8)
Single	28 (32.6)
Education level	
High school graduate	33 (38.4)
Undergraduate	37 (43.0)
Graduate	16 (18.6)
Employment status	
Retired	10 (11.6)
Unemployed	30 (34.9)
Employed	46 (53.5)
Socioeconomic status	
High	17 (19.8)
Middle	56 (65.1)
Low	13 (15.1)
Concurrent ADM intake	
No	18 (20.9)
Yes	68 (79.1)
Number of previous ADM trials	1.8 [0.9]

Note. ADM = antidepressant medication.

Adapted from R. Heshmati, F.J. Wienicke, E. Driessen, The effects of intensive short-term dynamic psychotherapy on depressive symptoms, negative affect, and emotional repression in single treatment-resistant depression: A randomized controlled trial, Psychother. 60 (2023) 497–511. https://doi.org/10.1037/pst0000500.

Copyright 2023 by the American Psychological Association.

## 4 Experimental Design, Materials and Methods

Participant recruitment took place in Tabriz, Iran, from April to May 2020. Individuals were referred from the waitlists of four clinical psychologists and four outpatient mental health clinics.

Eligibility criteria constituted: age between 18 and 60 years, a minimum of high school education, and a diagnosis of major depressive disorder according to the Diagnostic and Statistical Manual of Mental Disorders (4th edition) [12]. Diagnoses were established using the Mini International Neuropsychiatric Interview–Plus [13], conducted by two trained raters. Treatment resistance was defined as non-response to at least one adequate trial of antidepressant medication, in the context of a current depressive episode that had persisted for six weeks or longer. Antidepressant use during the trial was permitted if the dosage remained stable.

Exclusion criteria were: the presence of a comorbid personality disorder, psychotic or bipolar depression, psychotic spectrum disorders, substance dependence, cognitive impairments, suicidality or self-injurious behavior, and serious medical conditions such as cardiovascular disease, diabetes, or neurological trauma. Patients who had received psychotherapy in the preceding year or were unable to commit to weekly sessions were also excluded.

Eligible participants were randomly assigned in a 1:1 ratio to ISTDP or waitlist using a computer-generated randomization list. Those assigned to ISTDP received 20 individual sessions for 10 weeks according to the procedures described by Davanloo [1].

Data were collected before treatment, at post-treatment (10 weeks), and at 3-month follow-up. Two self-report outcome measures were administered at each time point: the Weinberger Adjustment Inventory (WAI) [14] and the Positive and Negative Affect Schedule (PANAS) [16] Negative Affect subscale.

The WAI includes 81 items assessing emotional and behavioral adjustment. It comprises three scales and ten subscales:

• Distress scale (29 items), consisting of four subscales: anxiety (8 items), depression (7 items), low self-esteem (7 items), and low well-being (7 items).
• Restraint scale (30 items), consisting of four subscales: suppression of aggression (7 items), impulse control (8 items), consideration of others (7 items), and responsibility (8 items).
• Defensiveness scale (22 items), consisting of two subscales: repressive defensiveness (11 items), and denial of distress (11 items)

Items include statements such as “I feel so down and unhappy that nothing makes me feel much better” (depression subscale) or “I never act like I know more about something that I really do” (repressive defensiveness subscale), which are rated on a 5-point Likert scale ranging from 1 = “False/Almost Never” to 5 = “True/Always”. Subscale scores are obtained by summing the corresponding items, and scale scores are calculated by summing the relevant subscale scores. Additionally, a Repressive/Restraint Composite Score can be computed by dividing the restraint scale score by three and then adding the repressive defensiveness subscale score [14]. The internal consistency of the subscales has been reported as questionable to good (α = 0.68 to 0.89) [15].

The PANAS negative affect subscale comprises 10 items that measure experiences of negative affect. Respondents indicate to what extent they have felt a certain way over the past week, e.g., scared, hostile, irritable. Items are rated on a 5-point Likert scale ranging from 1 = “Very slightly or not at all” to 5 = “Extremely”. The negative affect subscale score is calculated by summing up the 10 items. Research supported the external validity of the subscale and indicated good internal consistency (α = 0.87) [15].

Before the start of treatment, participants reported their demographic information (age, gender, marital status, education level, employment status, and socioeconomic status) and two clinical characteristics (prior treatment history and concurrent antidepressant use).

## Limitations

Not applicable

## Ethics Statement

Informed consent for participation in the trial was obtained from all participants, and ethical approval for the randomized controlled trial was granted by the Research Ethics Committee of the University of Tabriz (Approval code: IR.TABRIZU.REC.1400.012). In addition, the Research Ethics Committee of the University of Tabriz approved the deposition of the de-identified dataset in a public research data repository (Approval code: not applicable).

## CRediT Author Statement

**Rasoul Heshmati:** Conceptualization, Investigation, Project Administration, Data Curation, Methodology, Writing-Review & Editing. **Frederik Wienicke:** Formal Analysis, Data Curation, Writing-Original Draft. **Ellen Driessen:** Supervision, Writing-Review & Editing.

## Data Availability

OSF)RCT Dataset ISTDP vs. Waitlist for Treatment-Resistant Depression.sav (Original data)
